# Tissue Engineering and Its Potential to Reduce Prostate Cancer Treatment Sequelae—Narrative Review

**DOI:** 10.3389/fsurg.2021.644057

**Published:** 2021-10-14

**Authors:** Jan Adamowicz, Luis Alex Kluth, Marta Pokrywczynska, Tomasz Drewa

**Affiliations:** ^1^Chair of Urology, Department of Regenerative Medicine, Collegium Medicum, Nicolaus Copernicus University, Bydgoszcz, Poland; ^2^Department of Urology, University Hospital Frankfurt, Frankfurt am Main, Germany

**Keywords:** prostate cancer, stem cell, tissue engineering, urology, incontenence

## Abstract

Tissue engineering offers the possibility to overcome limitations of current management for postprostatectomy incontinence and ED. Developed in recent years biotechnological feasibility of mesenchymal stem cell isolation, *in vitro* cultivation and implantation became the basis for new cell-based therapies oriented to induce regeneration of adult tissue. The perspective to offer patients suffering from post-prostatectomy incontinence or erectile dysfunction minimal invasive one-time procedure utilizing autologous stem cell transplantation is desired management.

## Introduction

Prostatectomy is recommended choice of treatment for localized disease while in advanced cases the indications for surgery are gradually extending (1). The leading disadvantage of prostatectomy are side effects such as incontinence and erectile dysfunction (ED), still occurring despite continued progress in surgery technic (2). High number of patients recover from incontinence after rehabilitation but 10–20% suffer from persistent incontinence and 20–70% from erectile dysfunction (3). GLOBOCAN 2018 estimated, 1,276,106 new cases of prostate cancer were reported worldwide in 2018, with the highest prevalence in the developed^1^. In consequence, the correspondingly large number of patients suffering from prostatectomy side effects is generated each year. Approximately 50 and 30% of patients seek some form of treatment for incontinence and ED following prostatectomy, respectively (4). Modern Urology offers obviously management options for these patients. Nevertheless, a conservative approach has limited efficiency, and invasive forms of treatment including implantation of male slings, artificial urinary sphincters, or penile prosthesis need to be performed in most cases (5).

Tissue engineering offers the possibility to overcome limitations of current management for postprostatectomy incontinence and ED. Developed in recent years biotechnological feasibility of mesenchymal stem cell isolation, *in vitro* cultivation and implantation became the basis for new cell-based therapies oriented to induce regeneration of adult tissue (6). The perspective to offer patients suffering from postprostatectomy incontinence of ED minimal invasive one-time procedure utilizing autologous stem cell transplantation is a tempting idea. In this concept stem^1^ cells are intended to induce partial regeneration of sphincter complex and neuronal network damaged during surgery what would mediate functional recovery. In this short narrative review, we are presenting current research data focused on tissue engineering strategies addressing incontinence and ED after prostatectomy.

## Consequences of Iatrogenic Injury After Prostatectomy

Despite spectacular technical advances including magnification, 3D viewing, instrument miniaturization, and computer control of movement provided by the DaVinci system, prostatectomy is still an invasive procedure. This surgery has a damaging effect on crucial continence mechanism function in a significant percentage of patients regardless of the used method (7). Reported incontinence rates after prostatectomy can be as high as 80%. First of all, the proximal sphincteric unit is completely removed and in addition, the proximal urethral sphincter is damaged during prostate apex resection. Ultimately, this destructive cascade of events results in that postoperative continence depends largely on the rhabdosphincter (8). In contrast, intact male continence mechanism relies on the coordinated interplay of the inner lissosphincter of smooth muscle and an outer rhabdosphincter of skeletal muscle (9). Notwithstanding this ambiguous morphological characteristic of the male sphincter complex, the continence primarily depends on the proper lissosphincter activity (10). The internal sphincter controls passive continence and holds urine at the level of the vesical orifice. The synchronized contraction of its circular muscle fibers closes the vesical orifice and triggers concentric narrowing of the posterior urethra. Most importantly, the proper function of the lissosphincter is enough to guarantee passive continence (11). In this scenario, rhabdosphincter acts as a supporting component responsible for voluntary continence control. The nerve supply of the vesicourethral smooth muscle descends from the hypogastric and pelvic nerves for sympathetic and parasympathetic supply, respectively (12). In contrast, the rhabdosphincter receives somatomotor innervation from the pudendal nerve. Although the gross anatomy of sphincter complex innervation is well determined the localization of the intramural branched neuronal network within lower urinary tracts is still a matter of discussion. The cavernous nerve runs as a distinct bundle structure only in 30% of patients, whereas 70% have been shown to have plate architecture (13). Eichelberg et al. demonstrated that the most periprostatic nerves contributing to cavernous nerve were found posterolaterally but a significant portion of the nerves (22–29%) was located on the anterior surface of the prostate (14). Cavernous nerve terminations originating from the pelvic plexus release nitric oxide during sexual stimulation that leads to the relaxation of the smooth muscle fibers of the arteries and arterioles of the erectile tissue. The dogmatic location of prostate neurovascular bundles (NVBs) within the posterolateral aspect was confirmed using male cadavers only in approximately half the cases (15). In fact, NVBs exposed proximally dispersed fan-shape running course widely embracing anterior prostate plate (16). Independent of the applied surgical technique, prostate mobilization causes multifocal neuropraxia of the neural plexus mainly in the dorsal prostatic capsule. Additionally, some of the collaterals running from the pudendal nerve might have been unintentionally intersected. The development of the posterior plane between the prostate and rectum results in an unintentional mechanical disruption of the thin neuronal network mainly within Denonvilliers fascia (17). Applied traction during surgical maneuvers in multilayer environments generates shear force responsible for neuronal injury (18). It might be one of the explanations of failures in the nerve sparing approach. As a result of prostatectomy, the heterogenic injury occurs involving unpredictable denervation and structural damage of sphincter complex mainly of lissosphincter running from bladder neck through surrounding prostatic and membranous urethra (19). In addition, vesicourethral anastomosis creates a new anatomical spatial configuration of urine outflow and in fact continence mechanism now on the bladder neck and remaining membranous urethra (20). Therefore, well-supported vesicourethral anastomosis remains crucial for anastomotic healing after radical prostatectomy.

The lesion after prostatectomy encompasses reaming sphincter and neuronal plexus. Developing inflammation includes the release of transforming growth factor β1 (TGF-β1), platelet-derived growth factor (PDGF), tumor necrosis factor-α (TNF-α), and interleukin-1 (IL-1) (21). In the case of the urinary tract wall, activated urothelial cells become cell population that regulates the early phase of the inflammatory response. Their important role is to stimulate muscle precursors to responsive proliferation and maturing (22). Additionally, on the tissue level the inflammatory response triggers edema, acidosis, and apoptosis, which extend the injury site beyond vesicourethral anastomosis (23). The postoperative local hypoxia upregulates TGF-β/Smad signaling being a major profibrotic pathway (24). As a result, the gradual increasing accumulation of type I and III collagens within the sphincter muscles takes place leading to disruption of its architecture impairing bladder neck closure (25). A contractile, scar may in time overgrew sphincter muscle component causing its impartment even if the neuronal supply is functional. First 3 months after prostatectomy is defined as the acute phase of the injury and thereafter the most efficient improvement in terms of continence and erectile function occurs (26). This time period corresponds to the early remodeling phase ending with developed initial scar tissue and ended neuronal regeneration (Wallerian degeneration) (27). From the physiological perspective improvement after this time is rather related to rehabilitation or adaptive mechanism rather than active regeneration *per se*.

An important deterioration factor of regeneration is the environment of urinary tracts making them vulnerable to prolonging inflammation sustained by urine and microbiological contamination (28). Dovi et al. (29) demonstrated that a deletion of polymorphonuclear leukocyte (PMN) results in acceleration of wound closure. Chronic or excessive inflammation promotes scar formation and hamper tissue mechanisms to repair. Furthermore, the persistence of urine within the healing anastomosis is an often underestimated factor that may have a negative effect on final anastomosis remodeling. It was demonstrated that urine has a cytotoxic effect on muscles precursor cells participating in urinary tract wall regeneration (30). The primary approach of tissue engineering should aim to transform heeling pattern within the vesicourethral anastomosis and adjacent tissues.

## Tissue Engineering Approach

Healing is a highly evolved defense mechanism against infection and further injury. Adult human healing in lower urinary tracts is mediated mainly by a fibroproliferative response leading to scar formation (31). In contrast, urothelium as typical for epithelium characterizes with spontaneous regeneration capacity. Tissue engineering utilizes biomaterials and stem cells to induce intrinsic regeneration mechanisms that were silenced during ontogenesis. Healing of the urinary tract wall is initially led by activated urothelial cells that trigger the formation of the active subpopulation of mesenchymal precursors cells within the muscle layer (32). The signaling pathways including (Shh, Wnt, and Bmp) are upregulated during this process analogously to organogenesis stages (33). Building upon recent progress in understanding the molecular background of the healing process, tissue engineering focuses on controlled modulation of the healing milieu, thus resulting in a more favorable regeneration.

Most stem cells used in induced urinary tract regeneration are bone marrow-derived mesenchymal stem cells (BMSCs) containing significant proliferative capacity, long-term self-renewal potential, and having the ability to differentiate into other lineages. These stem cell populations exhibited high plasticity potential and were able to differentiate into urothelium and muscle layer *in vitro* under defined culture conditions (34). Therefore, delivered mesenchymal stem cells act as a source of paracrine signaling molecules acting on nearby cells. BMSCs are involved in all three phases during the wound-healing process. They also may enhance wound healing by immune modulation, production of growth factors that boost neovascularization, and reepithelialization (35). Nevertheless, the harvesting procedure of BMSCs is invasive for the patients and expensive. For this reason, although BMSCs are considered as a gold standard for adult stem cells, adipose-derived stem cells (ADSCs) gained considerable attention as a suitable candidate to be used in future therapies for patients after prostatectomy (36). ADSCs are characterized by less expensive cost of harvesting, greater yield, and confirmed multilineage differentiation ability that is the same as BMSCs. Zuk et al. (37) demonstrated the efficient capacity for myocyte differentiation *in vitro* when cultured next to myoblasts. Myocyte obtained from ADSCs could repair myotubes of ischemic muscular injury. Fakhrieh et al. (38) demonstrated that ADSCs could be a source of urinary bladder smooth muscle cells.

At the beginning of tissue engineering research, the dominant belief was that implanted stem cells locally replace injured tissue by direct differentiation and forming incorporated neotissue. At present, however, we are of the opinion that the regeneration effect is a result of realizing bioactive molecules (6). In particular, paracrine stimulation of angiogenesis is of utmost importance as it is a major profibrotic factor. ADSCs were documented to mediate angiogenesis by releasing growth factors including VEGF, HGF, and basic fibroblast growth factor (bFGF) (39). Chen et al. demonstrated that ADSCs are involved in cross-talk between endothelial cells, muscle precursors, and ECM during angiogenesis (40). ADSCs promoted endothelial colony-forming cell proliferation and differentiation. Interestingly, they could also differentiate into pericytes to stabilize the newly formed vessel structure.

At present, experimental attempts to modify the healing response by targeting individual pathways were not effective due to still insufficient knowledge about intricate signaling networks. Accordingly, ADSCs play the role of natural carriers of bioactive substances realized in an efficient way including timing, dosage, and interaction profile. Pokrywczyńska et al. (41) demonstrated, that ADSCs initiated regeneration of bladder wall mainly by the upregulation of the Hedgehog signaling pathway. Molecular analysis proved that implanted ADSCs activated cardinal pathways including GF-β, Jak-STAT, PI3-Akt, and Hippo governing early stages of urinary tract organogenesis.

The significant limitation of stem cell therapies utilizing *in vivo* cell implantation is a very low survival rate (<5%) (42). Although MSCs are considered as immune-privileged due to the absence of MHC-II expression, *in vivo* testing showed that MSCs upregulate MHC-II expression at the inflammation site and can be recognized by the host immune system (43). Uncontrolled stem activity characterizes with rather a low efficiency as these cells cannot *per se* rebuild damaged structures. Hence, all types of stem cells demand guiding signals to achieve the therapeutic effect (44). In these circumstances, tissue engineering may offer solutions and needed technology. Constructing cell implantable seeded grafts with a 3D biomaterial scaffold may offer the ability to precisely deliver cells into the injury site. This approach would also allow the creation of a temporary stable and supportive environment to gain time for the stem cells to impact the local paracrine milieu.

## Neuroregeneration

Stimulation of neuronal network regeneration mediating continence and erectile function after prostatectomy is the most challenging task awaiting to be addressed in future studies. Tissue engineering attempts to apply stem cells transplantation to reconstitute damaged intramural neuronal network (45). Conducted research showed that MSCs modulated neuroregeneration events including the Wallerian degeneration stage, accelerating remyelination, increasing neurofilament number, and enhancing fiber organization (46). Nevertheless, these results were observed using isolated peripheral nerve gap models that are not adequate to draw conclusions for potential regeneration of intramural convolutional neural network within the urogenital tract (47, 48). The targeted regeneration of neuronal network resected during prostatectomy is at present out of range of current biotechnology (28). Based on available research data MSCs contributed to neuronal regeneration by supplying the healing environment with neuroprotective bioactive factors including nerve growth factor (NGF), brain-derived neurotrophic factor (BDNF), and neurotrophin (49). This effect was achieved by direct MSCs paracrine activity and indirectly by acting on the Schwann cells (50). There are only several studies evaluating the ability of MSCs to induce neuromuscular regeneration by delivering cells in the neighborhood of damaged nerves (51). MSCs proved feasibility to stimulate neuronal ingrowth, elongation, and restoring neuronal network (52). We need to keep in mind that the injury site after prostatectomy is a particularly adverse environment with disrupted anatomical and histological structure. Hypothetically, it would be more rational to apply hybrid cellular-biomaterial systems rather than untargeted stem cell implantation. Combing stem cells with biomaterial corresponding to tissue-engineered bypass planned to bridge transected neuronal bundles during prostatectomy may be an interesting pathway to explore. Taking into account individually variable innervation within the prostate, we could design personalized bypass graft based on mapping of periprostatic neurons using, for instance diffusion tensor magnetic resonance (53). An unorthodox solution could be also using the autologous Schwann cells intended to exert local neuroprotective effect and stimulate neuroregeneration (54).

## Vascular Regeneration

During prostatectomy, it is necessary to transect or ligate branches of the pudendal artery, prostatic vesical bundles, and Santorini's plexus. These steps alter the blood supply to the vesicourethral anastomosis region and the penile structures, mainly corpus cavernosa (55). A major clinical manifestation of these circulation disturbances is susceptibility to vesicourethral stenosis and regressive morphological changes in the corpus cavernosa. Although the mechanism of vesicourethral stenosis is poorly understood, it involves two main parallel events, namely, uncontrolled expansion of the muscle layer and a fibrosing reaction promoted by hypoxic environment (23). Underlying inflammation and hypoxic environment only intensify this chronic process. Analogously, progressive fibrosis takes place in the corpora cavernosa after prostatectomy as denervation and chronic ischemia (56).

Mesenchymal stem cells may be utilized in cell-based therapy to support angiogenesis of healing thereby providing potentially therapeutic benefits after prostatectomy. The lesson learned from the field of cardiology exposed the ability of MSCs to actively migrate to ischemic areas after myocardial infarction. Mesenchymal cells improved remodeling of the infraction zone by inducing transmyocardial revascularization (57). Stem cell-based therapies aimed to improve functional results after prostatectomy need to promote regional postoperative angiogenesis both within the remaining of the sphincter and corpus cavernosa. The secretome of the MSCs includes proangiogenic factors extracellular vesicles (EV) carrying miRNAs (58). Regardless of the tissue of origin, enrichment of miRNAs in MSC-EVs has been shown to promote angiogenesis *in vitro* and *in vivo*. miRNAs originated from MSCs targeted the expression of regulatory angiogenic genes encoding for cytokines, MMPs, VEGF, PDGF, fibroblast growth factor (FGF), and epidermal growth factor (EGF) (59). Several miRNAs with angiogenic potential such as miRNA-494, miR-125a, or miR-210 were described in MSC-derived EVs (60).

## Stem Cell Safety

The safety of stem cells therapies is one of the major concerns of clinicians, especially in oncological patients. The major risk is related to the use of pluripotent embryonic cells that exposed the highest self-renewal potential and differentiation capacity. There are reports describing tumor formation after autologous multipotent stems cell transplantation (61). Particular attention should be also paid to the fact that current models of cell therapy can require hundreds of millions of cells per patient, which need to be expanded *in vitro*. Adaptation of self-renewing cells to their culture conditions poses the risk of latent cancerogenesis (62). Regardless of applied argumentation it must be underlined that the real risk of iatrogenic tumor formation after stem cell implantation within solid organs is not clearly determined. The situation becomes even more controversial if we plan to deliver stem cells in the neighborhood of the malignant tumor resection zone. In addition to the tumorigenic potential inherent to differentiation capacity, the direct influence of the remaining cancer cells is another possible hazard. It was shown that MSC-derived exosomes can promote tumor growth through a variety of mechanisms (63). The wide profile of stem cells secretome might act as a two-edged sword in this scenario. Therefore, the same bioactive molecules can simultaneously and advantageously modify the healing environment and promote cancer recurrence. However, MSC-derived exosomes were found to exhibit an inhibitory effect on prostate cancer, so this cell population seems to be particularly suitable for urological application (64). From the other hand, the in-depth interplay between MSCs and prostate cancer cells has not been established. In light of the postulated MSC involvement in the development of androgen-independent prostate cancer, the utilization of this cell population should be only limited to patients who do not pose a risk of recurrence (65).

## Postprostatectomy Incontinence

To date, five clinical trials aimed to evaluate cell therapy for postprostatectomy stress incontinence were completed (Table 1). The first study evaluating cell-based therapy for urinary incontinence after prostatectomy was published by Mitterberger et al. (66). In this study, 63 patients with stress urinary incontinence after radical prostatectomy were treated with transurethral ultrasound-guided injections of autologous fibroblasts and myoblasts obtained from skeletal muscle biopsies. The applied combination of cellular populations was intended to act bilaterally. Accordingly, fibroblasts were aimed to counteract atrophy of submucosa within the urethra to improve the passive selling mechanism of the remaining supra-membranous urethra. Whereas, implanted myoblast was planned to contribute actively to rhabdosphincter remodeling by increasing the number of contractile fibers. After 12 months of follow-up, 58 patients showed relevant improvement. Although the authors provided multicriteria analysis to evaluate therapy success using subjective and objective tools, the main limitation of the study is the low number of patients and the lack of a control group. In consequence, there is no possibility to discriminate between the effects of spontaneous regeneration and the results of guided remodeling of the cells. On the other hand, the study was distinguished by a large number of implanted cells that were precisely administered with invented ultrasound guided system. Gerullis et al. presented data from a one arm study, in which male patients with stress urinary incontinence (including 197 after prostatectomy) were treated with a transurethral injection of autologous muscle-derived cells (67). Transurethral implanted cells were at least 50% of myogenic origin and predominantly represented early stages of muscle cell differentiation. The authors demonstrated an improvement in 42% of patients. Only patients with endoscopically proven sphincter damage were included. However, the limitation of the study is the non-standardized inclusion criteria, resulting in a heterogeneous cohort. Moreover, endoscopically visible sphincter dysfunction is an indirect sign of rather a severe sphincter injury that is unlikely to be repaired with the most basic form of cell therapy. Accordingly, a medium form of stress incontinence seems to be the most adequate for a cell-based approach, which should not be categorized as an ultima ratio or alternative for artificial sphincter.

**Table 1:** Cell therapy clinical trials for stress urinary incontinence after prostatectomy.

**Study**	**Number of patients**	**Time after prostatectomy**	**Type of cells**	**Evaluation tools**	**Administration method**	**Administrated cells**	**Patients with reported improvement (%)**
Mitterberger et al. (66)	63	Min. 12 mths Avg. 43 mths	Fibroblasts Myoblasts	Incontinence score I-QOL Transurethral US Urodynamics	US guided endoscopic transurethral injection	Avg. 3.8 × 10^7^ fibroblasts Avg. 2.8 × 10^7^ myoblasts	58 (92%)
Gerullis et al. (67)	222	Min. 12 mths	MDC	“In-house” continence questionnaire	Endoscopic transurethral injection	Avg. 5.2 × 10^6^	90 (41%)
Gotoh et al. (68)	11	12 mths	ADRC	24h pad test Urodynamics ICIQ-SF MRI	Endoscopic periurethral injection	Avg. 1.8 × 10^7^	8 (70%)
Choi et al. (69)	6	12 mths	ADRC	24h pad test Urodynamics ICIQ-SF MRI	Endoscopic periurethral injection	(no data)	6 (100%)
Garcia-Arranz et al. (70)	9	Avg. 60.5 mths	ADSC	24h pad test Urodynamics ICIQ-SF SF-36	Endoscopic periurethral injection	2 × 10^6^ (2 patients) 6 × 10^6^ (8 patients)	8 (88%)

*MDC, Muscle-derived cells; ADRCs, Adipose-derived regenerative cells; ADSCs, adipose-derived stem cells; I-QOL, urinary incontinence quality of life scale; US, ultrasound; ICIQ-SF, international consultation on incontinence questionnaire—Short Form; SF-36, 36 item short form survey*.

**Figure 1:**
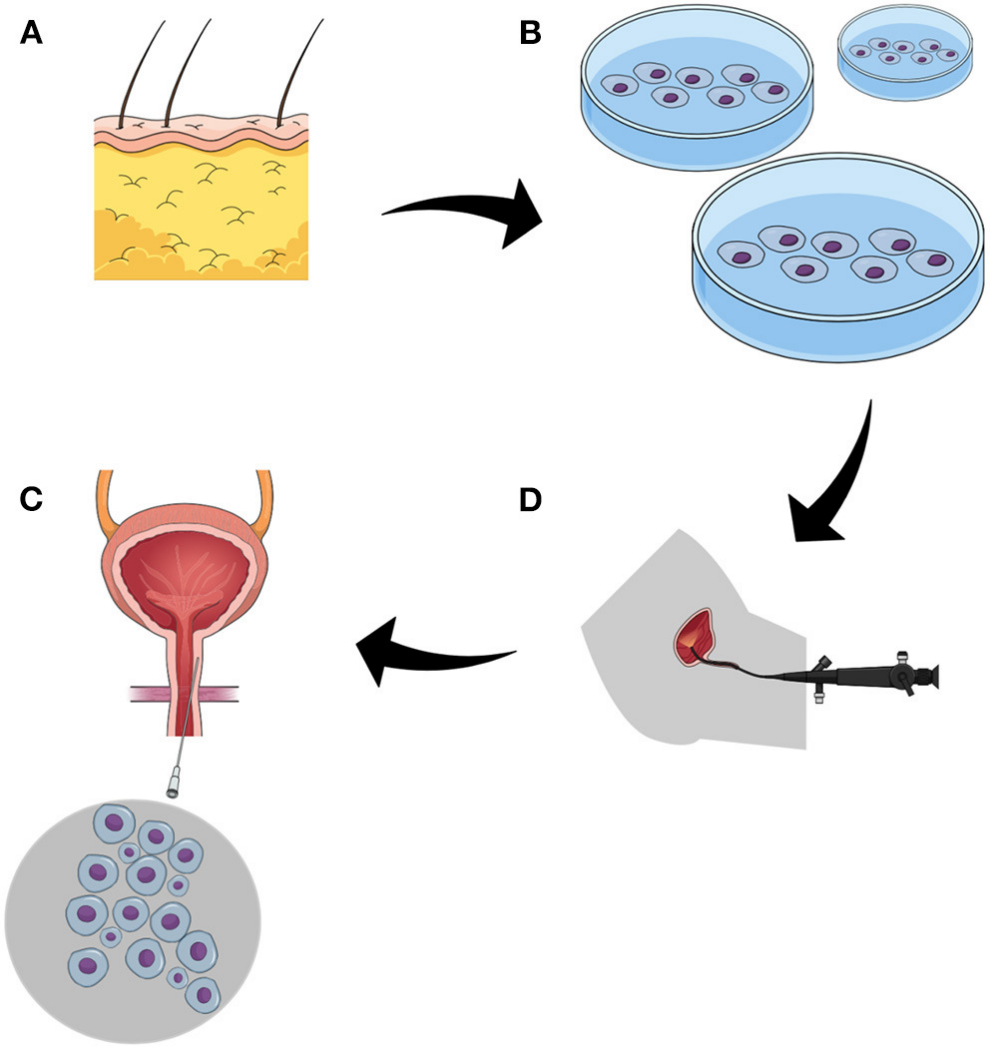
The figure demonstrates the strategy of cell-based approach to postprostatectomy stress urinary incontinence. **(A)** adipose tissue, the source of mesenchymal stem cells. **(B)** isolated stem cells underwent purification and propagation *in vitro*. **(C)** the function of the sphincter is evaluated endoscopically, the most common approach utilizes flexible urethroscopy. **(D)** administration of stem cells to sphincter complex by targeted multiple injections.

Following early studies utilizing mature adult cells, the concept evolved into using MSCs offering the potential to induce natural regeneration (Figure 1). Gotoh et al. were the first to introduce the concept of using adipose-derived regenerative cells (ADRCs) from abdominal adipose tissue obtained by liposuction (68). ADRCs are a heterogeneous population of cells including multipotent adipose-derived stems cells, other progenitor cells, fibroblasts, T-regulatory cells, and macrophages. In this setting, ADRCs obtained by the Celuton system were suspended in untreated lipoaspirate and transurethrally injected into the rhabdosphincter and submucosal space in 11 patients. Stress urinary incontinence improved in eight patients during 1 year of follow-up. The authors evaluated therapy success by urethral closing pressure and functional profile length, which were both significantly elevated. Although adipose-derived stem cells have the capacity to differentiate into contractile cells, no evidence demonstrating potential incorporation of implanted cells with host sphincter structure was provided. Moreover, the major concerns arose after analyzing the volume of injected material. In total each patient received apart from direct injection of 1 ml ADRCs, 20 ml of relatively a thick suspension of lipoaspirate with the narrow region of the external urethral sphincter. In this situation, it is highly probable that the observed impairment was the result of persisting bulking effect. Indeed, the authors discussed this potential problem but did not rule out this possibility by creating a control group. Based on acquired data the same group registered in the 2015 ADRESU study claimed to be the first clinical trial of regenerative treatment for stress urinary incontinence by ADRCs (71). The primary endpoint of the ADRESU study is to be urine leakage volume reduction from baseline >50% by the 24-h pad test at 52 weeks. In 2016, Choi et al. inspired by Gotoh conducted a clinical trial using the same protocol in six patients (69). Although the study was not bringing any new insight authors showed feasibility to replicate efficacy and safety of stem cell therapy for incontinence. Application of the commercially available cell-processing Celution system allowed to obtain standardized ready-to-use cell suspension. This is a role model of how modern stem cell-based therapy in the field of urology should look like. Recently, Garcia-Arranz et al. (70) demonstrated results of the first nonrandomized phase I–IIa clinical trial involving nine men after prostatectomy. The tested feasibility of using ADSCs injected in the region of the bladder neck and along the external sphincter under visual guidance using compact cystoscope guidance. Overall, 38% of patients showed an objective clinical improvement of more than 50% which is in line with the FDA definition of optimal continence improvement after therapeutic intervention. In two of the eight patients, continence improvement was noticed after initial administration of 20× 10^6^ cells. In the rest of the patients, the second dose, according to the study protocol, was necessary. Administration protocol of the cells including multiple cell injections into the injured sphincter is likely to be more effective in supplying regeneration environment with bioactive molecules. The studies aimed to induce regeneration of ischemic heart exposed a very low MSC survival rate after transplantation. Similarly, the harsh microenvironment of injured sphincter with ischemia, inflammation, oxidative stress, and mechanical stress contributes to great cell loss shortly after administration. It is the rationale for developing administration protocols with several injection time schedules. Despite the low number of patients, the study of the Madrid group is so far the most advanced and complex report from the field of experimental cell-based therapy for urinary incontinence after prostatectomy. The analysis of the clinical trials database also provides information on the newly launched trial in Belarus (NCT04446884). As stated in brief, the recruitment for treatment of urinary incontinence in men after with autologous ADSCs was launched in June 2020. The summary of the achievements to date in the treatment of urinary incontinence with autologous cell implantation indicates that the field is in early clinical research phase I.

All studies are characterized by low patient numbers and the omission of control group. The inclusion of the control group should be of utmost importance in future trials as postprostatectomy incontinence improves spontaneously in an individual and difficult to predict manner. Another unknown parameter is the number of cells mandatory to obtain a therapeutic effect. Administrated cell numbers varied between studies, and more importantly, this issue was not comprehensively discussed. The choice of the number of cells in a given therapy is rather empirical and mostly depended on the efficiency of the isolation method. Indeed, we do not know what the best stem cell number is to improve continence or erectile function. In all studies, sphincter regeneration or remodeling guided by implanted cells remained within speculation. Alleged revascularization and neuronal or mesodermal regeneration were not objectively demonstrated, especially on the histological level. In this situation, it may be reasonable to focus on cell behavior after implantation using *in vitro* models or cell tracing techniques. Basic research with a purely cognitive focus is needed in this field to optimize trial protocols in terms of clinical and cost-efficiency. From a safety point of view, the demonstrated cell therapies did not have adverse effects reducing their usefulness. Importantly, liposuction needed for both cell harvesting and transurethral cell administration was well-tolerated procedures. Implantation of cells with high proliferation capacity and differentiation potential outside their normal niche may be a matter of concern in terms of local tumorogenesis. Gotoh et al. (68) conducted extensive follow-up with magnetic resonance imaging conducted every 3 months and could not observe any tumorogenesis within the injection site. The major question, however, remains in regard to the timing of the cell therapy initiation. In all clinical attempts, cells were delivered at least 1 year after prostatectomy, which clearly contradicts our understanding of the induced regeneration mechanism. Namely, at this time point, the scar tissue within the sphincter complex was already developed with silenced remodeling phase making the environment of vesicourethral anastomosis rather non-susceptible to induction of regeneration. It is also the lesson learned from clinical trials from the field of spinal cord injury where the results improved by reducing the time to administration of cells. There is a need to plan a trail where cells would be delivered shortly after the first PSA testing. The concerns related to boosting of resection area with bioactive molecules must be, however, taking into account. Raj et al. (72) explained the possible link between mesenchymal stem cells and prostate cancer progression risk. On the other hand, the in-depth understanding of prostate cancer biology allows us to choose patients with local diseases with extremely low chances for recurrence after prostatectomy. Adequately, these patients with low and medium incontinence, ideally after nerve-sparing prostatectomy, should be the target population. Alternative strategies to stem cells implantation developed to ameliorate prostatectomy functional outcomes include grafts from the dehydrated human amniotic membrane (AM). Patel et al. were the first to present this method in 2015 (73). In the introduced technic AM was wrapped around the neurovascular bundle to improve healing. Reported results indicated that thanks to AM the recovery time for continence was significantly accelerated. AM is a naturally derived biomaterial containing over 226 different growth factors, cytokines, chemokines, protease inhibitors, and other bioactive molecules capable of modulating tissue healing (74). For this reason, AM is widely used in the field of ophthalmology to obtain scarless corneal healing. A significant reduction in the progression and severity of fibrosis was observed after using AM on demanding animal and clinical models. AM is gradually gaining popularity among Urologists, Barski et al. (75) described recently the design of a randomized, single-blind, placebo-controlled, phase 2 study of the efficacy and safety of AM during radical prostatectomy. An important advantage of AM is its natural high elasticity and eligibility during surgical procedures. It acts as a natural carrier of bioactive substances that could be placed in the neighborhood of neural bundles and pelvic plexus. AM was successfully evaluated for nerve bridge repair of peripheral nerve defects in animal models. These inexpensive and easy to obtain biomaterials is rich in cytokines and neurotrophic factors creating a suitable micro-environment for axonal regeneration (76).

## Erectile Dysfunction

Various degrees of cavernous nerve damage always occur during prostatectomy and even nerve-sparing surgery is no exception. Apart from mechanical injury of the pelvic plexus and its branches postprostatectomy, ED is a result of developing fibrosis due to prolonging penile flaccidity (77). The desired effects of potential stem cell-based therapy are expected to reverse the structural changes leading to ED and to mitigate patient dependence on the transitory effect of PDE5 inhibitors. Three clinical trials addressing the feasibility of using stem cell therapy in patients with ED have been completed so far (Table 2) (78–80). Applied subpopulations of mesenchymal stem cells were derived from multiple sources including bone marrow and adipose tissues. In all the cases, straightforward intracavernous stem cell administration was a well-tolerated procedure without relevant side effects and impact on prostate cancer follow-up. The available reports showed improvement in penile hemodynamics and cumulative erectile function scores. It is important to notice that Haahr et al. divided patients in terms of continence coexisting with ED and suggested that applied stem treatment might have a positive effect on incontinence *per se*. Nevertheless, major limitations included a low number of patients and a lack of standardized protocols, making the outcomes of the study difficult to compare and objectively judging the effectiveness of the therapy. The mechanism of stem cell action after extracavernous administration was also hypothetically formulated. The postulated regenerative effect was achieved by either secreting growth factors locally boosting cavernous tissue or by ascending migration to the pelvis plexus and supporting neuronal regeneration on ganglion level. There is a lack of evidence that implanted stem cells generate replacement structures of erectile incorporated with the native one. Despite current limitations and still unanswered questions, stem cell-based therapy for patients after prostatectomy is offered in the private medical sector (81). However, it must be underlined that its clinical suitability is still unknown and must be assessed by clinical trials.

**Table 2:** Cell therapy clinical trials for erectile dysfunction after prostatectomy.

**Study**	**Number of patients**	**Time after prostatectomy**	**Type of cells**	**Evaluation tools**	**Administration method**	**Administered cells**	**Patients with reported improvement (%)**
Yiou et al. (78)	12	6 mths to 3 yr	BM-MNC	IIEF-15 EHS Doppler US	Intracavernous injection	2 × 10^7^; 2 × 10^8^ 1 × 10^9^; 2 × 10^9^	12 (100%)
Yiou et al. (79)	12	6 mths to 3 yr	BM-MNC	IIEF-15 EHS Doppler US	Intracavernous injection	1 × 10^9^	12 (100%)
Haahr et al. (80)	17	5–18 mths	ADRC	IIEF-5 EHS	Intracavernous injection	9 × 10^6^	8 (47%)

*BM-MNC, Bone-marrow derived mononuclear cell; ADRCs, Adipose-derived regenerative cells; IIEF-15, International Index of Erectile Function-15; EHS, Erectile Hardness Score; IIEF-15, International Index of Erectile Function-5*.

## Conclusions

Tissue engineering has an unquestionable potential to improve the current management of postprostatectomy stress incontinence and erectile dysfunction. Conducted studies provided clues that remodeling of the injured sphincter complex could be induced by stem cells. Similarly, erectile tissue was regenerated by implanted stem cells. These methods are so far the most advanced therapeutical options for patients that do not compensate action of impaired structures but try to restore proper function. Nevertheless, none of the conducted studies has enough translational potential to reliably introduce these types of therapies into clinical practice. The still unanswered questions regarding the most optimal time schedule of therapy, regenerating cell population, administration method, and advantage over the available pharmacological treatment need to be addressed in future trials.

## Author Contributions

JA: concept and writing. LK: concept and corrections. MP and TD: supervision. All authors contributed to the article and approved the submitted version.

## Conflict of Interest

The authors declare that the research was conducted in the absence of any commercial or financial relationships that could be construed as a potential conflict of interest.

## Publisher's Note

All claims expressed in this article are solely those of the authors and do not necessarily represent those of their affiliated organizations, or those of the publisher, the editors and the reviewers. Any product that may be evaluated in this article, or claim that may be made by its manufacturer, is not guaranteed or endorsed by the publisher.

## Footnotes

^1^https://gco.iarc.fr (accessed February 9, 2020).
